# Cognitive reserve in Older Adults with Bipolar Disorder and its relationship with cognitive performance and psychosocial functioning

**DOI:** 10.1192/j.eurpsy.2024.672

**Published:** 2024-08-27

**Authors:** L. Montejo, C. Torrent, S. Martín, A. Ruiz, M. Bort, G. Fico, V. Oliva, M. De Prisco, J. Sanchez-Moreno, E. Jimenez, A. Martinez-Aran, E. Vieta, B. Sole

**Affiliations:** ^1^Bipolar and Depressive Disorders Unit, Hospital Clinic of Barcelona, CIBERSAM, IDIBAPS, University of Barcelona; ^2^Bipolar and Depressive Disorders Unit, Hospital Clinic of Barcelona, IDIBAPS, University of Barcelona; ^3^Bipolar and Depressive Disorders Unit, Hospital Clinic of Barcelona, CIBERSAM, IDIBAPS, Departament de Medicina, Facultat de Medicina i Ciències de la Salut, University of Barcelona, Barcelona, Spain

## Abstract

**Introduction:**

Cognitive reserve (CR) refers to the ability of the brain to cope with damage or pathology. In bipolar disorder (BD), it has been seen that the effects of the disease may potentially reduce CR, thus compromising cognitive outcomes. This concept takes on special relevance in late life in BD, due to the increased risk of cognitive decline because of the accumulative effects of the disease and the potential effects of aging. Therefore, we believe that CR may be a protective factor against cognitive decline in older adults with bipolar disorder (OABD).

**Objectives:**

The aim of this study was to study the CR in OABD compared with healthy controls (HC) and to analyze its association with psychosocial functioning and cognitive performance.

**Methods:**

A sample of euthymic OABD, defined as patients over 50 years old, and HC were included. CR was assessed using the CRASH scale. Differences in demographic, clinical, and cognitive variables between patients and HC were analyzed by t-test or X2 as appropriated. Lineal simple and multiple regressions analyses were used to study the association of CR and several clinical variables with functional and cognitive performance.

**Results:**

A total of 83 participants (42 OABD and 41 HC) were included. Compared to HC, OABD exhibited poorer cognitive performance (p<0.001), psychosocial functioning (p<0.001) and lower CR (p<0.001). Within the patient’s group, the linear simple regression analysis revealed that CR was associated with psychosocial functioning (β=-2.16; p=0.037), attention (β= 3.03; p=0.005) and working memory (β = 2.98; p=0.005) while no clinical factors were associated. Age and CR were associated with processing speed and verbal memory, but after applying multiple regression model, only the effect of age remained significant (β =-2.26; p= 0.030, and β =-2.23; p= 0.032 respectively). CR, age, and number of episodes were related to visual memory, but the multiple regression showed that only age (β = -2.37; p= 0.023) and CR (β = 3.99; p<0.001) were associated. Regarding executive functions only the number of manic episodes were significant. CR and age at onset were associated with visuospatial ability, but multiple regression only showed association of CR (β =2.23; p=0.032). Other clinical factors such as number of depressive or hypomanic episodes, illness duration, admissions, type of BD, and psychotic symptoms were not associated.

**Conclusions:**

To the best of our knowledge, this is the first report that studies the CR in a sample of OABD. We demonstrated that OABD had lower CR than HC. Importantly, we observed that CR was associated with cognitive and psychosocial functioning in OABD, even more than disease-related factors. These results suggest the potential protector effect of CR against cognitive impairment, supporting that improving modifiable factors associated with the enhancement of CR can prevent cognitive decline.

**Disclosure of Interest:**

L. Montejo: None Declared, C. Torrent Grant / Research support from: Spanish Ministry of Science and Innovation (PI20/00344) integrated into the Plan Nacional de I+D+I and co-financed by the ISCIII-Subdireccion General de Evaluacio ´n and the Fondo Europeo de Desarrollo Regional (FEDER), S. Martín: None Declared, A. Ruiz: None Declared, M. Bort: None Declared, G. Fico Grant / Research support from: Fellowship from “La Caixa” Foundation (ID 100010434 - fellowship code LCF/BQ/DR21/11880019), V. Oliva: None Declared, M. De Prisco: None Declared, J. Sanchez-Moreno Grant / Research support from: Spanish Ministry of Science and Innovation (PI20/00060) integrated into the Plan Nacional de I+D+I and co-financed by the ISCIII-Subdireccion General de Evaluacio ´n and the Fondo Europeo de Desarrollo Regional (FEDER),, E. Jimenez Grant / Research support from: Spanish Ministry of Science and Innovation (PI20/00060)integrated into the Plan Nacional de I+D+I and co-financed by the ISCIII-Subdireccion General de Evaluacio ´n and the Fondo Europeo de Desarrollo Regional (FEDER),, A. Martinez-Aran: None Declared, E. Vieta Grant / Research support from: Spanish Ministry of Science and Innovation (PI18/ 00805, PI21/00787) integrated into the Plan Nacional de I+D+I and cofinanced by the ISCIIISubdireccio ´n General de Evaluacio ´n and the Fondo Europeo de Desarrollo Regional (FEDER); the Instituto de Salud Carlos III; the CIBER of Mental Health (CIBERSAM); the Secretaria d’Universitats i Recerca del Departament d’Economia i Coneixement (2017 SGR 1365), the CERCA Programme, and the Departament de Salut de la Generalitat de Catalunya for the PERIS grant SLT006/17/00357; the European Union Horizon 2020 research and innovation program (EU.3.1.1. Understanding health, wellbeing and disease: Grant No 754907 and EU.3.1.3. Treating and managing disease: Grant No 945151)., B. Sole: None Declared

